# Infection in Joint Arthroplasty: Diagnosis, Prevention, and Treatment Strategies—A Comprehensive Narrative Review

**DOI:** 10.3390/life15121853

**Published:** 2025-12-02

**Authors:** Jovana Grupkovic, Miroslav Ceculovic, Uros Dabetic, Dejan Aleksandric, Nikola Bogosavljevic, Ranko Lazovic, Slavisa Zagorac

**Affiliations:** 1 Clinic for Orthopedic Surgery and Traumatology, University Clinical Center of Serbia, 11000 Belgrade, Serbia; ceculovicmiroslav@gmail.com (M.C.); urosdabetic1983@gmail.com (U.D.); slavisa.zagorac@gmail.com (S.Z.); 2 Faculty of Medicine, University of Belgrade, 11000 Belgrade, Serbia; boga19@gmail.com; 3 Institute for Orthopedic Surgery “Banjica”, 11000 Belgrade, Serbia; aleksandricdejan@gmail.com; 4 Special Surgical Hospital “A3 Medical”, 85355 Sutomore, Montenegro; ranko.lazovic5@gmail.com; 5 Montenegrin Academy of Scencies and Arts, 81000 Podgorica, Montenegro

**Keywords:** antimicrobial therapy, arthroplasty infection, biofilm, diagnosis, infection prevention, periprosthetic joint infection, revision arthroplasty, surgical debridement, treatment strategies

## Abstract

Background: Periprosthetic joint infection (PJI) remains one of the most severe complications after total joint arthroplasty, causing significant morbidity and healthcare burden. Despite advances in surgical techniques, diagnostics, and antimicrobial therapy, infection rates have not declined substantially, emphasizing the need for comprehensive preventive and therapeutic strategies. Methods: This narrative review synthesizes evidence from peer-reviewed clinical studies, consensus statements, and major international guidelines addressing periprosthetic joint infection in hip, knee, and shoulder arthroplasty. Results: Recent evidence highlights advances in diagnostic biomarkers, molecular testing, and standardized consensus criteria that have improved early detection. Surgical strategies such as DAIR, one-stage, and two-stage revisions—combined with biofilm-active antimicrobial therapy—remain the mainstay of management. Preventive measures focusing on perioperative optimization, infection control, and antibiotic stewardship are the most effective means to reduce infection risk. Conclusions: Future progress relies on precision-based prevention, novel biofilm-targeted therapies, and multidisciplinary collaboration to achieve durable, infection-free outcomes following joint arthroplasty.

## 1. Introduction

Periprosthetic joint infection (PJI) and surgical site infections (SSIs) represent two of the most severe and costly complications in modern orthopedic surgery. Although total joint arthroplasty (TJA) is among the most successful interventions in medicine, capable of dramatically improving pain, mobility, and quality of life, infection remains a persistent threat to long-term outcomes.

Periprosthetic joint infection affects approximately 0.5–2% of primary arthroplasties and remains a leading cause of early failure, with PJI accounting for a substantial proportion of revision procedures [1,2,3].

As populations age and life expectancy increases, the global demand for joint replacement surgery—particularly total hip arthroplasty (THA) and total knee arthroplasty (TKA)—continues to rise. A substantial increase in the number of patients diagnosed with PJIs is expected in the coming years due to the increasing volume of total joint arthroplasties performed internationally [2,3].

PJI is a biologically distinct and clinically complex entity. Unlike acute surgical site infections, PJIs often present subacutely or chronically and are characterized by the formation of bacterial biofilms on the surface of the implant and surrounding tissue. PJI is associated with significant patient morbidity, increased rates of revision procedures, prolonged hospitalization, and substantial financial burden on healthcare systems.

The diagnosis of PJI is itself a formidable challenge. Clinical presentations can range from overt wound drainage and systemic sepsis to subtle pain and stiffness without obvious signs of infection. Conventional diagnostic tools such as C-reactive protein (CRP) and erythrocyte sedimentation rate (ESR) lack specificity, particularly in low-grade or indolent infections. Optimization and development of efficient protocols for managing PJIs can lead to the establishment of preventive measures and the most effective diagnostic methods based on the results obtained from laboratory tests [4,5]. Over the past two decades, several international working groups, including the Musculoskeletal Infection Society (MSIS) [6], the Infectious Diseases Society of America (IDSA) [7], and the International Consensus Meeting on Musculoskeletal Infection (ICM) [8], have proposed diagnostic and therapeutic guidelines to aid clinicians in the complex decision-making required for managing PJI.

Treatment options for PJI vary widely depending on timing, host status, pathogen virulence, and previous surgeries.

Acute postoperative infections occurring within the first four weeks after surgery may be amenable to DAIR (debridement, antibiotics, and implant retention), whereas delayed infections developing between 4 weeks and 24 months, and late hematogenous infections presenting beyond 24 months, more often require prosthesis removal and staged reimplantation.

The purpose of this narrative review is to synthesize contemporary evidence on the epidemiology, microbiology, diagnosis, prevention, and treatment of periprosthetic joint infection in hip, knee, and shoulder arthroplasty. By integrating current clinical data, consensus guidelines, and emerging research, this review aims to provide a comprehensive and clinically relevant overview that supports multidisciplinary decision-making and highlights areas requiring further investigation.

## 2. Materials and Methods

This narrative review synthesizes peer-reviewed evidence relevant to the epidemiology, diagnosis, prevention, and treatment of periprosthetic joint infection (PJI). Articles were selected based on clinical relevance, quality of evidence, and contribution to current understanding of PJI. Priority was given to meta-analyses, consensus guidelines, and high-quality cohort studies, while case reports and basic science papers were included selectively when clinically illustrative. Sources were identified through targeted keyword searches and manual review of reference lists. A brief summary of the scope of evidence considered is provided in Table 1.

## 3. Epidemiology of Periprosthetic Joint Infection

Early and late failure of both total hip arthroplasty (THA) and total knee arthroplasty (TKA) can be attributed to PJIs and also surgical site infections (SSIs). Epidemiological data from large national joint registries show that PJI accounts for approximately 15–25% of revision total knee arthroplasties and 10–15% of revision total hip arthroplasties [9]. Although in primary arthroplasty revision rates remain low—ranging from 0.5% to 2%, depending on joint type and institution—these figures translate into a substantial absolute number of cases given the increasing global volume of joint replacements [10].

The number of THA and TKA procedures is expected to grow; in the U.S. only, the number of PJI cases is projected to exceed 270,000 by 2030, which is an alarming issue [11,12]. Demographic patterns of PJI vary considerably according to geographic region, healthcare infrastructure, and registry methodology. In high-income settings, where perioperative care and infection surveillance are standardized, most infections present early in the postoperative period [13]. Conversely, in regions with limited healthcare resources, chronic and late-presenting infections are more common, reflecting gaps in prophylaxis and delayed recognition. Shoulder arthroplasty-related infections, although less prevalent than those involving the hip or knee, reveal distinctive epidemiological trends [14].

## 4. Microbiology of Periprosthetic Joint Infection

Periprosthetic joint infections present a unique microbiological profile compared to typical skin or soft-tissue infections. A prosthesis adds complexity by altering host–pathogen interactions and promoting biofilm formation on its surface. Accurate pathogen identification underpins effective treatment strategies.

A great number of PJIs is caused by Gram-positive cocci, especially staphylococcal species. Over 50% of infections are caused by Staphylococcus aureus, both methicillin-sensitive (MSSA) and methicillin-resistant (MRSA), and coagulase-negative staphylococci (CoNS) such as Staphylococcus epidermidis [15]. Such organisms readily form biofilms, making infections especially persistent and difficult to clear once they have attached to prosthetic implants.

Other notable pathogens include Enterococci, particularly *Enterococcus faecalis*, Streptococcal species, Gram-negative bacilli including *Escherichia coli*, *Pseudomonas aeruginosa*, and *Klebsiella pneumoniae*, and anaerobes such as *Cutibacterium acnes* [16].

### 4.1. Biofilm Formation and Its Clinical Implications

The key difference that sets PJIs apart from other infections is the presence of biofilm. Biofilm is a complex of microorganisms embedded within an extracellular matrix of polysaccharides, proteins, and nucleic acids, and serves as a fundamental mechanism of organism survival and persistence in PJI. It is impairing the activity of many antimicrobials by altering local pH and impacting microbial metabolic activity and replication [17].

Biofilm formation occurs in the following several stages:

1. **Initial adhesion** of planktonic (free-floating) bacteria to the implant surface.
2. **Microcolony formation**, involving bacterial proliferation and production of extracellular matrix.
3. **Maturation**, where the biofilm develops a complex architecture with channels for nutrient flow.
4. **Dispersion**, in which planktonic bacteria are released to colonize other areas.

Once the biofilm is formed, bacterial activity slows down, allowing them to enter a dormant phase where they become extremely resistant, up to 1000 times more resistant to antibiotics than planktonic forms [18]. This necessitates the use of biofilm-active antibiotics, such as rifampin (for *staphylococci*) or combination therapy, and often requires surgical removal of the implant for definitive cure.

Clinical consequences of biofilms are as follows:

- Reduced efficacy of standard culture techniques due to the bacterial dormant phase;
- Increased rates of treatment failure and infection recurrence;
- Necessity of prolonged antibiotic therapy (typically ≥6 weeks);
- Influence on surgical decision-making: implants harboring biofilms may require removal even if appearing macroscopically intact.

### 4.2. Culture-Negative PJI

Culture-negative PJI is still a diagnostic and therapeutic dilemma. Reported in up to 20% of cases, it is attributed to the following:

· Prior antibiotic use before sample collection;
· Low-virulence or fastidious organisms;
· Inadequate sampling techniques or laboratory conditions.

Diagnosis is largely guided by clinical judgment, histopathological findings, and non-culture-based biomarkers (e.g., synovial α-defensin, leukocyte esterase). In these scenarios, empirical antimicrobial therapy is typically initiated, often targeting staphylococci and Gram-negatives until better identification is achieved [19].

Biofilm makes identification of pathogens more difficult, as it hides the pathogen during joint aspiration. Also, it is not always possible to collect multiple samples safely from the same joint, as it is necessary to avoid false-negative results.

With increasing globalization, widespread antibiotic utilization, and an aging global population, uncommon pathogens are being reported with greater frequency, they are as follows:

· Fungal PJIs, most commonly *Candida albicans*, typically occurring in immunocompromised patients or after prolonged antibiotic exposure [20].
· Mycobacterial infections, such as *Mycobacterium tuberculosis*, which can mimic aseptic loosening and present with chronic symptoms.
· Multidrug-resistant organisms (MDROs), particularly in high-risk settings or revision surgeries, which pose significant challenges for both medical and surgical management.
· Zoonotic and environmental pathogens, like Brucella or Nocardia, which, although rare, should be considered in appropriate epidemiological contexts.

### 4.3. Polymicrobial Infections

Polymicrobial PJIs are defined as the isolation of two or more causative microorganisms. Approximately 10–20% of PJIs are polymicrobial, usually involving staphylococci, streptococci, and Gram-negative rods. These infections are associated with poorer outcomes, largely due to increased diagnostic complexity, broader resistance profiles, and challenges in selecting effective antibiotic combinations [21]. 

These infections are particularly common in the following patients:

· Patients with prior wound complications;
· Post-traumatic arthroplasty;
· Immunocompromised hosts or those with multiple reoperations.

Key risk factors for polymicrobial infections are the presence of a sinus tract, soft-tissue defects, obesity, and prior revision procedures.

### 4.4. Advances in Microbial Identification

Recent advancements are improving pathogen detection and may reduce the incidence of culture-negative cases:

· Sonication of explanted implants, which helps dislodge biofilm-embedded organisms, has increased diagnostic yield by 10–30% compared to standard tissue cultures [22].
· Molecular techniques, such as polymerase chain reaction (PCR), 16S rRNA sequencing, and metagenomic next-generation sequencing (mNGS), are increasingly available in specialized centers.
· Matrix-assisted laser desorption ionization–time of flight (MALDI-TOF) mass spectrometry allows for rapid species identification but is still dependent on initial culture.

Despite these innovations, barriers such as high cost, need for standardization, and limited availability restrict their widespread use in everyday clinical practice.

## 5. Diagnosis of Periprosthetic Joint Infection

Diagnosing periprosthetic joint infections is a complex and often time-consuming process. Acute infections may present with clear clinical signs like fever, joint pain, erythema, or purulent drainage, but many PJIs, particularly those caused by low-virulence organisms, manifest with subtle or non-specific symptoms. Delayed or chronic infections often mimic aseptic loosening, which may lead to misdiagnosis and inappropriate treatment. Early and accurate identification of PJIs is of great importance, as delays can impair outcomes and limit the success of implant-retaining strategies. The timing of presentation also influences the underlying microbiology: early postoperative infections (<4 weeks) are more often caused by virulent organisms such as *Staphylococcus aureus*, whereas delayed and late infections frequently involve low-virulence pathogens including coagulase-negative staphylococci or *Cutibacterium acnes* [15,16].

Because there is no single definitive test for PJI, diagnosis relies on a combination of clinical, laboratory, microbiological, histological, and imaging findings, structured through validated diagnostic criteria. Several major international bodies have proposed and refined diagnostic guidelines, including Musculoskeletal Infection Society (MSIS) [6], International Consensus Meeting (ICM) [8], and the European Bone and Joint Infection Society (EBJIS) [23] (Table 2).

Initial evaluation begins with a thorough history and physical examination. Key elements include the following:

· Timing of symptoms relative to index arthroplasty (early < 3 months, delayed 3–24 months, late > 24 months).
· Presence of fever, joint pain, wound drainage, or presence of sinus tract.
· History of recent bacteremia, dental procedures, or prior surgical site infections.

In chronic infections, symptoms may be subtle, limited to mechanical loosening or mild joint discomfort. In such cases, diagnosis relies heavily on laboratory and synovial findings.

Serological markers remain widely used in initial screening for PJI, but they lack specificity and may be mistaken for systemic inflammation or comorbidities. CRP (C-reactive protein) is commonly used, elevated levels may suggest presence of infection, but it is not specific. ESR (erythrocyte sedimentation rate) can be elevated (>30 mm/h); it peaks later and declines more slowly. Interleukin-6 is more specific than CRP in acute infections, but it is not yet standardized.

Joint aspiration and synovial fluid analysis are considered the cornerstone of preoperative diagnosis, particularly in chronic cases. Ideally, joint aspiration and synovial fluid analysis are performed after withholding antibiotics for two weeks. Administration of prophylactic antibiotics at induction does not significantly reduce intraoperative culture yield when meticulous sampling is performed, although prolonged or repeated antibiotic exposure before surgery can lower detection rates, particularly in low-grade infections [4].

Synovial biomarkers have high sensitivity and specificity because they are measured directly in the synovial fluid of the suspected joint [24]. They commonly include IL-6, IL-7, IL-17, TNF, and synovial CRP [25]. Also, several more sensitive and specific biomarkers have been evaluated in the diagnosis of PJI, such as alpha-defensin [26], cathelicidin LL-37, human beta-defensins 2 and 3 [27], leukocyte esterase [28], and calprotectin [29]. 

Alpha-defensin is a synovial biomarker used in PJI diagnosis, which has a higher specificity than synovial CRP. It is an immune-type reaction that measures alpha-defensin concentration from synovial fluid. Alpha-defensin is an antimicrobial peptide secreted by human cells in response to the presence of pathogenic bacteria [30]. Its role is to integrate into the cell membrane of the pathogenic bacterium, leading to its rapid destruction.

Leukocyte esterase (LE) is another biomarker used to diagnose PJI. It is an enzyme secreted by activated neutrophils, with high levels especially in patients with different urological conditions. In PJI, neutrophils that reach the joint following infection produce LE, that can be detected using colorimetric tests based on a color-changing reaction [31]. 

Another biomarker analyzed in PJI is calprotectin. The calprotectin identification test is an immune reaction that measures calprotectin concentration in the synovial fluid [32]. This protein is a pro-inflammatory factor of innate immunity that activates toll-like receptor (TLR) four and is released by activated granulocytes during inflammatory processes [33]. It has been shown to have 100% sensitivity and a specificity of 95% in PJI diagnosis [34].

Each biomarker has specific advantages and limitations. Alpha-defensin is highly accurate and unaffected by antibiotic exposure, but its high cost limits widespread use. Leukocyte esterase is inexpensive and provides rapid results, though colorimetric interpretation may be subjective and less reliable in bloody aspirates. Calprotectin offers high sensitivity and specificity at low cost, but its availability is still limited in many centers. In clinical practice, alpha-defensin is mainly used in equivocal cases, leukocyte esterase as a rapid screening tool, and calprotectin as an emerging alternative where available.

Imaging is adjunct to laboratory workup, especially when aspiration is non-diagnostic or contraindicated.

· Plain radiographs may show osteolysis, periosteal reaction, or implant loosening; sensitivity is low.
· Nuclear imaging is useful in chronic or culture-negative cases.
· MRI and CT are limited by metallic artifact but increasingly useful with metal artifact reduction sequences (MARS-MRI).
· FDG-PET/CT is promising for detecting low-grade infection but is not yet universally accepted due to cost and limited access [35].

In recent years, molecular diagnostics have positively impacted microorganism identification. Molecular techniques such as polymerase chain reactions (PCRs) and next-generation sequencing (NGS) can be used for detecting pathogenic bacteria in cultures. PCR can detect pathogens in synovial fluid with a sensitivity of 84% and a specificity of 89% [36]. Although these molecular technologies offer improved pathogen detection, they also present important limitations. PCR and 16S rRNA sequencing may detect non-viable organisms or contaminants, which can complicate interpretation. Metagenomic next-generation sequencing (mNGS) is highly sensitive but costly, not widely available, and prone to environmental or reagent contamination. These methods currently complement, rather than replace, conventional cultures, and their clinical utility often depends on institutional resources and expertise (Table 3).

## 6. Prevention Strategies

Prevention is still the most effective and cost-efficient approach to managing PJI. Preventive measures can be broadly classified into modifiable and non-modifiable risk factors. Modifiable factors include smoking, poor glycemic control, obesity, malnutrition, and *Staphylococcus aureus* colonization [10,37,38,39,40,41], all of which have strong to moderate supporting evidence from cohort studies and guideline recommendations. Non-modifiable factors, such as advanced age, prior revisions, inflammatory arthritis, and underlying immunosuppression [10,37,38,39,40], also contribute to risk, although the supporting evidence is more heterogeneous and generally less robust. Recognizing the differing strengths of evidence behind each category allows clinicians to prioritize interventions that offer the greatest potential to reduce infection risk.

While surgical treatment requires extensive resources and multidisciplinary care, many infections can be avoided if we stick to evidence-based protocols across all phases of the surgical pathway. Chronologically, these protocols can be applied during the preoperative, intraoperative, and postoperative stages.

### 6.1. Preoperative Measures

Several patient-related factors that predispose to PJI can be modified in the preoperative stage. An extensive preoperative assessment should include screening for the following:

· Glycemic control: Aim for Hgb A1c < 7.5%; avoid surgery if >8.0% [42].
· Nutritional status: Address hypoalbuminemia (<3.5 g/dL) or vitamin deficiencies.
· Smoking cessation: Recommended at least four weeks prior to surgery.
· Weight reduction: Morbid obesity (BMI > 40) is an independent risk, so delaying surgery until weight reduction should be considered.

Decolonization: Screen for nasal *Staphylococcus aureus* carriage. In many institutions, extended screening including axilla and groin swabs is recommended to improve detection of *Staphylococcus aureus* or MRSA colonization, as these sites may serve as additional reservoirs.

Diabetes mellitus (DMs) is increasing in prevalence, and it is estimated to affect approximately 9.3% of the adult population worldwide, and is projected to grow further [43]. DM is closely related to a higher likelihood of surgical site infections, especially uncontrolled diabetes (>200 mg/L or Hgb A1c > 7%), by amplifying postoperative complication risk. The American Diabetes Association recommends delaying surgery for those with an HbA1c greater than 7% [44]. The long-term effects of hyperglycemia compromise the immune system, and microangiopathic changes impede wound healing in diabetic patients. It is highly recommended to maintain perioperative blood glucose concentrations between 110 and 180 mg/dL, facilitated by routine monitoring and adept postoperative diabetic management protocols [45].

Smoking negatively impacts surgical outcomes through several mechanisms. Nicotine, the primary harmful component of tobacco, has been linked to microvascular constriction and decreased oxygen delivery to tissues, impairing wound healing and increasing the likelihood of post-surgical infections [37]. Given that smokers undergoing TJA have shown significantly higher rates of surgical site infection compared to non-smokers [38], smoking cessation programs have been highly recommended.

Current guidelines recommend complete smoking cessation at least four weeks before arthroplasty to reduce the risk of wound complications and PJI. Continued abstinence in the postoperative period is also advised, as nicotine exposure impairs tissue oxygenation and delays healing, further increasing infection risk [37,38].

The increasing number of obese patients presents a critical challenge in arthroplasty. Patients undergoing TJA are especially vulnerable to risks associated with obesity. Studies have shown that a high body mass index (BMI) significantly elevates the risk of PJI, and morbid obesity (BMI > 40 kg/m^2^) increases the risk of periprosthetic joint infection by approximately 2–4 times compared with non-obese patients [10,39,40].

Some studies, particularly those evaluating high-risk TKA populations, have reported that morbid obesity may increase infection risk up to 21-fold [10], although most contemporary analyses demonstrate a more moderate 2–4-fold increase.

Obesity brings with it other health risks, like metabolic syndrome, wound dehiscence, and heart disease, that further complicate surgical outcomes. Because of these heightened risks, weight loss prior to TJA has been widely recommended; even a 5 to 10% weight reduction has been shown to provide cardiovascular and metabolic benefits [41]. 

### 6.2. Intraoperative Measures

The operating room environment is critical for infection control, as many pathogens are airborne and originate from the surgical team itself [46]. Therefore, several modifications to the OR environment have been examined, such as laminar airflow systems, high-efficiency particulate air (HEPA) filtration, the use of sterile body exhaust suits, and also reducing staff traffic and door openings during surgery. The effectiveness of laminar airflow systems in reducing bacterial load within the OR has been supported by multiple studies [46,47], holding promise for improved infection control, but the cost-effectiveness of these systems is debatable. Among available systems, vertical laminar airflow is generally considered more effective than horizontal flow because it provides a unidirectional downward air pattern that better isolates the surgical field, reducing contamination risk, although its clinical advantage remains debated.

Surgical technique and handling also affect PJI risk in patients. Shorter operative time (<120 min) correlates with reduced infection rates [48]. Aseptic technique, including double-gloving and minimal implant handling, is essential. Hand antisepsis is still the most cost-effective measure in the operating room, with both alcohol-based hand scrubs and water-based hand scrubs with certified antiseptics [49]. Usage of skin sealants and drapes at the incision site that are imbued with bacteriostatic agents shows protection against SSIs, but has been the subject of discussion [50]. Surgeons can significantly influence PJIs through their choices in wound closure techniques, operative speed and efficiency, and their careful tissue handling.

Intraoperative irrigation with dilute povidone-iodine or chlorhexidine is increasingly common and has been shown to reduce bacterial load. Many institutions use a 0.5% povidone-iodine solution as standard protocol due to its proven efficacy and low fibroblast toxicity [47,51]. 

Antibiotic prophylaxis is another crucial intraoperative measure. Administration of cefazoline (2 g IV or 3 g if >120 kg) within 60 min of incision is recommended [52]. Specific antibiotic selection should ultimately follow local antimicrobial resistance patterns and institutional guidelines, which may differ between regions and hospitals.

Regarding the duration of antibiotic therapy, most guidelines and studies support discontinuation within 24h post-surgery, as prolonged use does not seem to offer additional benefits [53]. Use of vancomycin in patients with a history of methicillin-resistant Staphylococcus aureus (MRSA) in dual-antibiotic prophylaxis is recommended [54]. 

Antibiotic-loaded bone cement is commonly used in both primary and revision arthroplasty to reduce the risk of PJI. Gentamicin or vancomycin are the most frequently incorporated agents. Its main advantages include high local antibiotic concentrations with minimal systemic toxicity; however, concerns remain regarding potential weakening of the cement matrix, selection of resistant organisms, and variability in elution characteristics. In clinical practice, antibiotic-loaded cement is routinely used in revision procedures and selectively in high-risk primary arthroplasties.

### 6.3. Postoperative Care

As for the postoperative period, there are some steps that should be followed. Wound dressings used should be occlusive and maintain a sterile barrier for 5–7 days. Minimize or avoid drain usage, and if possible, remove them within 24–48 h. Any drainage beyond 72 h warrants investigation.

## 7. Treatment Principles

Treatment of PJI is complex, and requires a multidisciplinary approach involving orthopedic surgeons, infectious disease specialists, microbiologists, and pharmacists. Success depends not only on pathogen eradication, but also on preservation of joint function, patient quality of life, and minimization of complications.

Once the diagnosis of PJI is suspected or confirmed, surgical plans are made. The ideal treatment strategy must consider the timing of infection, virulence of the causative organism, implant stability, condition of soft tissues, and host comorbidities. A clear understanding of treatment principles provides the foundation for selecting between implant retention, partial or full revision, and in some cases, salvage procedures.

There is more than one classification for PJIs, one of the most used was suggested by Coventry, who proposed three distinct stages of infection: the acute phase (in the first three months), phase 2 (more than three months after the operation), and phase 3 (two years after the previous infection of the joint) [55], as well as classification based on timing and onset, as shown in Table 4. Another intensively cited classification is the one formulated by Tsukayama et al., who proposed a classification system for periprosthetic infections consisting of the following four groups: positive intraoperative cultures, early postoperative infection occurring before four weeks, late chronic infection (>4 weeks), and acute hematogenous infection [56].

Goals of treatment are eradication of infection, by removing biofilm where necessary and delivering effective antimicrobial therapy; restoration of function, to preserve or reconstruct joint stability and mobility; avoidance of recurrence, to minimize re-infection risk with optimized host and implant conditions; and minimizing morbidity by tailoring interventions to patient frailty and preferences.

### Treatment Modalities

Debridement, antibiotics, and implant retention (DAIR) is the first line of treatment, typically used for acute and hematogenous infections, for well-fixed, functioning implants, and when a healthy soft-tissue envelope is present [57]. This procedure includes radical debridement and synovectomy, exchange of modular components, copious lavage, and prolonged culture-directed antibiotics (6–12 weeks). Success rates range from 30 to 80%, depending on pathogen virulence, surgical technique, and timing [58]. 

One-stage exchange arthroplasty is preferred in chronic PJI with known, sensitive organism, when there is adequate soft tissue and bone stock, and when the patient is capable of withstanding a longer initial procedure. In one-stage exchange arthroplasty, all device components are removed, and new revision components are inserted after debridement as part of the same surgical procedure.

Two-stage exchange arthroplasty is still considered the gold standard in many institutions and it is preferred when there is polymicrobial infection, culture-negative cases, a sinus tract, extensive bone loss, or soft-tissue compromise. In the first stage, all components are removed, and a temporary antibiotic-laden spacer device is placed. The second stage of definitive reimplantation is performed months later following the administration of systemic antibiotic therapy and confirmed infection resolution. The duration of antibiotic therapy before reimplantation is not uniform; although 6–12 weeks is common practice, the exact course depends on the pathogen, intraoperative findings, inflammatory marker trends, and institutional protocol.

Some newer studies and trials have shown that there is no difference in the re-infection rate between one-stage and two-stage revision THA for PJI [59]. Although both one-stage and two-stage revision strategies are well-established, the optimal approach remains debated. Evidence comparing the two is heterogeneous, with some studies suggesting similar infection-free survival, while others report higher eradication rates with two-stage revision in complex or resistant infections. The lack of standardized selection criteria and varying definitions of treatment success contribute to ongoing controversy [58,59].

Arthrodesis is rarely indicated in hip PJI but is more common in TKA when reimplantation is not possible. It offers pain relief and eradication of infection but results in loss of joint mobility.

Amputation is the last resort and it is reserved for life-threatening infection, massive bone/soft-tissue loss, or repeated failure of all other strategies.

**Figure 1:**
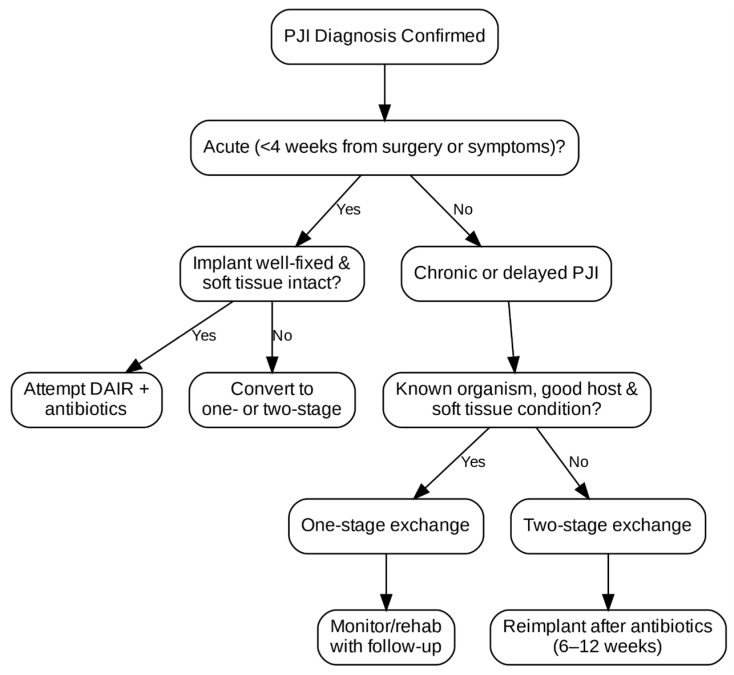
PJI surgical management flowchart.

Chronic suppressive antibiotic therapy may also be considered in high-risk patients with retained implants and persistent infection. It typically involves long-term oral antibiotics after IV induction. The goal of this kind of treatment is infection control, not eradication. It is controversial and reserved for nonoperative candidates or those who refuse surgery. The surgical management flowchart is shown in Figure 1.

## 8. Antimicrobial Therapy

Antibiotic therapy the is foundation of PJI management. Unlike many other infections, PJI presents unique challenges due to biofilm formation, altered vascularity near implants, and prolonged symptom duration before diagnosis. Because of that, antimicrobial treatment must be tailored not only to the pathogen and its resistance profile, but also to the timing, type of surgical intervention, and implant status.

Effective antimicrobial course requires close collaboration between infectious disease specialists, microbiologists, and orthopedic surgeons.

Empiric antimicrobial therapy is initiated immediately after cultures are drawn (ideally before surgery), and it should cover Gram-positive cocci, especially *Staphylococcus aureus* (including MRSA), and Gram-negative bacilli if the patient is immunocompromised or has prior revisions.

Common empiric regimens include the following:

· Vancomycin + Cefepime (broad-spectrum, MRSA + Gram-negatives);
· Vancomycin + Ceftazidime (alternative if cefepime not available);
· Vancomycin + Aztreonam (if penicillin allergy).

Once culture and susceptibility data are available, switch to targeted therapy, usually within 3–5 days.

For staphylococcal infections, most PJIs, therapy depends on methicillin susceptibility. In cases of MSSA, intravenous anti-staphylococcal beta-lactams such cefazolin, nafcillin, or oxacillin remain the first-line agents, administered for four to six weeks. For methicillin-resistant S. aureus (MRSA), vancomycin has traditionally been the preferred agent, though daptomycin has emerged as an effective alternative. Combination therapy with rifampin is strongly recommended whenever prosthetic material is retained, as rifampin exhibits unique biofilm activity, but it should never be used as monotherapy due to the rapid development of resistance [7]. As shown in Table 5, Table 6 and Table 7, most commonly found pathogens require combined antibiotic therapy.

Gram-negative organisms are as follows:

**Table 6:** Gram-negative organisms.

Pathogen	Preferred Antibiotics	Notes
*E. coli*, *Klebsiella* spp.	Ceftriaxone, Ceftazidime, Ertapenem	Duration: IV 4–6 weeks
*Pseudomonas aeruginosa*	Ceftazidime, Cefepime, Piperacillin–Tazobactam	May need combination therapy
MDR Gram-negatives	Colistin, Tigecycline, Meropenem–Vaborbactam	Reserve agents; use with ID guidance

Anaerobes and fungi are as follows:

**Table 7:** Anaerobes and fungi.

Pathogen	Preferred Antibiotics	Notes
*Cutibacterium acnes*	Penicillin G, Ceftriaxone	Often requires extended therapy; common in shoulders
*Candida albicans*	Fluconazole or Echinocandins	Requires implant removal in most cases

Rifampin is the cornerstone of biofilm-active therapy for staphylococcal PJIs, especially when the prosthesis is retained. Its use is associated with higher rates of eradication (Table 8).

The optimal duration and route of antimicrobial therapy in PJI depend largely on the surgical strategy employed and the extent of infection control achieved. Current evidence supports an initial course of intravenous antibiotics for four to six weeks in most cases, followed by tailored oral therapy when appropriate. The total treatment duration varies according to whether the implant is retained, exchanged, or permanently removed. As summarized in Table 9, patients managed with implant retention (DAIR) typically require prolonged combined therapy—usually six weeks of intravenous treatment followed by oral biofilm-active agents, such as rifampin combinations, for a total of approximately twelve weeks. One-stage and two-stage revisions generally follow shorter or intermediate regimens, depending on intraoperative cultures and host factors, while resection or arthrodesis cases may necessitate extended or indefinite oral suppression when reimplantation is not feasible. This individualized, procedure-specific approach balances the need for bacterial eradication with the goal of minimizing antibiotic toxicity and resistance.

### Oral Step-Down Therapy

The OVIVA trial showed that oral antibiotics (with adequate bioavailability) are non-inferior to IV therapy in bone and joint infections [60], including PJI, provided they fulfill the following conditions:

· The organism is known and susceptible.
· The patient is stable and adherent.
· An ID specialist is involved.

Common oral agents are as follows: rifampin, levofloxacin, linezolid, clindamycin, and trimethoprim–sulfamethoxazole.

## 9. Outcomes and Prognosis

PJI has substantial clinical and socioeconomic consequences. Even when diagnosis and appropriate treatment are timely, patients may experience multiple surgeries, prolonged antibiotic therapy, functional impairment, and reduced quality of life.

Success varies significantly by treatment strategy. The success rate for DAIR in acute cases is from 30% to 80%, and it depends on pathogen and the time when it is diagnosed [61]. For one-stage revision, success rate is from 85% to 90% in some cases [15]. Two-stage revision has an 85% to 95% success rate, and it remains the gold standard [62]. Resection arthroplasty and suppressive therapy have success rates from 60% to 70% but with poor function. These success rates derive from heterogeneous studies, including retrospective cohorts and meta-analyses with varying sample sizes, definitions of treatment success, and follow-up duration, which explains the wide range reported in the literature.

Key predictors of failure include the following:

· High-virulence organisms (e.g., MRSA, *P. aeruginosa*);
· Delayed or inadequate debridement;
· Poor soft-tissue coverage;
· Retained infected implants;
· Polymicrobial or culture-negative PJI.

Despite infection control, patients often report persistent joint stiffness or pain, reduced mobility, especially after two-stage procedures, lower return-to-work rates, and mental health impacts from prolonged treatment.

Recurrent PJI occurs in 5–15% of cases after revision. The risk is highest within the first two years post-reimplantation [63]. 

Several clinical scoring systems have been developed to aid in treatment selection and prognostic assessment of periprosthetic joint infection. As summarized in Table 10, the KLIC score helps predict failure after DAIR procedures by integrating key host and surgical factors such as renal or hepatic dysfunction, inflammatory markers, and cement use. The CRIME80 score focuses on elderly patients, identifying age, CRP, and infection characteristics as predictors of poor outcome. The McPherson classification remains a broader framework for risk stratification, incorporating host status, infection chronicity, and local conditions to guide individualized management strategies.

## 10. Challenges and Future Directions

Despite progress in PJI diagnosis and management, significant gaps remain in optimizing care. Multidisciplinary and technological advances continue to reshape the landscape of infection treatment.

Culture-negative PJIs still remain difficult to treat and study. Standardization of molecular techniques (e.g., NGS, PCR) is needed for widespread adoption. Also, a lack of universal consensus of definitions (MSIS vs. ICM vs. EBJIS) complicates research comparisons.

The increasing prevalence of multidrug-resistant organisms (MDROs) strains current therapeutic options. Global antibiotic management is critical to prevent resistance amplifications. There is a need for new biofilm-active antimicrobials with fewer toxicity limitations.

Next-generation sequencing and advanced biomarkers like metagenomic NGS (mNGS), exosomal microRNAs, and metabolic biomarkers are showing promise in diagnosing culture-negative or rare-pathogen PJIs, potentially enhancing early detection [64]. 

Antimicrobial photodynamic therapy (aPDT), utilizing photosensitizers activated by light to generate reactive oxygen species, shows potent microbial killing with minimal systemic resistance. Emerging materials enable localized pathogen control on demand [65].

Immune-modulating therapies—such as cytokine-targeted treatments—are under exploration to support host immunity and mitigate chronic inflammation, especially in drug-resistant or immunosuppressed populations [66].

Several emerging biofilm-targeted and implant-sparing therapies are in early clinical or preclinical development, and a brief overview is provided below for context (Table 11).

Continuous Local Antibiotic Perfusion (CLAP) delivers sustained high local antibiotic concentrations using negative-pressure gradients. It is promising for infection control, but concerns about cytotoxicity (e.g., gentamicin effects on osteoblasts) require further evaluation [67].

VT-X7 therapy is currently under study. It is a special device that delivers cyclical vancomycin and tobramycin antibiotics at sufficient concentrations to penetrate biofilm over seven-day period, after which prosthesis reimplantation and VT-X7 removal is performed. Also, one more innovation is PLG0206 (Peptilogics; Pittsburgh, PA, USA), a broad-spectrum anti-infective peptide which has shown promising preclinical activity against biofilm [68].

However, translating these innovations into standard practice faces the following hurdles: cost constraints, regulatory pathways, long-term safety data, and the need for multidisciplinary clinical trials.

Other promising antimicrobial strategies are also emerging in preclinical orthopedic research. Ceragenins, a class of cationic steroid antimicrobials, demonstrate broad-spectrum and biofilm-active properties while resisting enzymatic degradation, making them attractive for use in implant coatings and local delivery systems. In addition, bone-binding antimicrobials such as BBA-1, which chemically anchor to hydroxyapatite, offer the possibility of highly targeted infection control with reduced systemic toxicity. Although these approaches remain in early development, they highlight important future directions in implant-associated infection prevention.

## 11. Conclusions

Periprosthetic joint infection (PJI) remains one of the most challenging complications in contemporary arthroplasty, profoundly affecting patient function and quality of life. Although advances in surgical technique, perioperative optimization, and antimicrobial therapy have refined management, infection rates have not declined substantially, underscoring the complexity of host–pathogen interactions and biofilm biology.

Recent developments in molecular diagnostics, consensus criteria, and targeted antimicrobial protocols have improved diagnostic precision and therapeutic outcomes. Surgical approaches—ranging from implant retention to staged revision—must be individualized according to infection chronicity, microbial profile, and host status.

Looking ahead, prevention through meticulous perioperative optimization and adherence to standardized protocols remains paramount. The emergence of biofilm-active agents, local antibiotic delivery systems, and biofunctional implant coatings offers encouraging prospects for the future. Sustained multidisciplinary collaboration, together with robust translational and clinical research, will be essential to transform these innovations into truly infection-free arthroplasty.

## Figures and Tables

**Table 1:** Scope of evidence considered.

Category	Details
Evidence types prioritized	Meta-analyses, systematic reviews, consensus statements, cohort studies
Additional sources	Narrative reviews, expert guidelines, selected case series
Excluded	Non-peer-reviewed sources, non-relevant case reports, basic science without clinical correlation
Included study designs	Systematic and narrative reviews, cohort studies, consensus statements
Exclusion rationale	To ensure clinical relevance and data reliability

**Table 2:** Diagnostic criteria for PJI.

Criteria	MSIS (2018)	ICM (2018)	EBJIS (2021)
**Major Criteria**	Sinus tract communicating with prosthesis ≥2 positive cultures of same organism	Same as MSIS	Same as MSIS
**Minor Criteria**	Point-based scoring system: - CRP > 10 mg/L (2 pts) - Synovial WBC > 3000 (3 pts) - α-defensin positive (3 pts) - Positive histology (3 pts) - 1 positive culture (2 pts) - PMN > 80% (2 pts)	≥6 points = infection 4–5 = inconclusive <4 = no infection	Classified as follows: **Confirmed**, **Probable**, or **Possible** PJI Based on clinical, lab, histology, culture, and imaging
**Culture-negative PJI**	Possible if criteria met without culture	Acknowledged in scoring	Explicitly considered
**Strength**	Objective, widely adopted	Quantitative, nuanced	Inclusive, adaptable
**Weakness**	May miss low-grade infections	Complex in urgent settings	May over-diagnose infection

**Table 3:** Conventional vs. emerging diagnostic tests for PJI.

Test Type	Advantages	Limitations
**Conventional cultures**	Widely available; inexpensive; provides susceptibility testing	Low sensitivity in prior antibiotics; biofilm organisms may be missed
**PCR**	Rapid detection; useful for low-virulence organisms	Cannot distinguish live vs. dead bacteria; contamination risk
**16S rRNA sequencing**	Broad-range detection	Expensive; may detect contaminants; limited availability
**mNGS**	Detects rare/fastidious organisms; highly sensitive	Very high cost; long turnaround; contamination risk; limited access

**Table 4:** Classification based on timing and onset.

Type	Time Frame	Likely Pathogens	Clinical Features
**Early postoperative**	<4 weeks after surgery	*S. aureus*, CoNS	Redness, warmth, drainage
**Delayed**	4 weeks–24 months	Low-virulence organisms	Pain, stiffness, minimal signs
**Late (hematogenous)**	>24 months post-op	Often *S. aureus*, *Streptococci*	Acute onset, fever, painful joint

**Table 5:** Gram-positive organisms.

Pathogen	Preferred Antibiotics	Notes
*MSSA*	Nafcillin, Cefazolin, Oxacillin	IV 4–6 weeks; high cure rates
*MRSA*	Vancomycin or Daptomycin	Add Rifampin if implant retained
Coagulase-negative staphylococci	Vancomycin ± Rifampin	Rifampin must never be used as monotherapy
*Enterococcus faecalis*	Ampicillin ± Gentamicin	Requires synergy; resistance testing needed
*Enterococcus faecium* (VRE)	Linezolid or Daptomycin	Toxicity limits prolonged use

**Table 8:** Biofilm-active agents.

Agent	Use	Cautions
Rifampin	Add-on for MRSA/MSSA	Never use as monotherapy (resistance risk)
Daptomycin	MRSA, VRE	Monitor CPK levels (risk of myopathy)
Linezolid	MRSA, VRE	Risk of cytopenias with >14 days use
Fosfomycin	Used in combination	Investigational in orthopedic biofilm use

**Table 9:** Duration of therapy [6,7,8].

Surgical Strategy	IV Duration	Oral Therapy	Total Duration
DAIR	4–6 weeks	Yes (Rifampin combo)	12 weeks
One-stage exchange	4–6 weeks	±Oral tail	6–12 weeks
Two-stage exchange	4–6 weeks	No (usually)	Stop after reimplant
Resection/Arthrodesis	6 weeks	±Oral	Often indefinite if no reimplant planned

**Table 10:** Scoring tools.

Score	Purpose	Variables Included
KLIC Score	Predict DAIR failure	Kidney, liver, index surgery, CRP, cement
CRIME80 Score	Stratify failure in elderly	Age > 80, CRP > 150, male sex, infection type
McPherson Classification	Risk stratification	Host grade, infection type, local factors

**Table 11:** Emerging therapies in PJI: mechanisms and stage of development.

Therapy	Mechanism	Stage of Development	Clinical Relevance
**CLAP (Continuous Local Antibiotic Perfusion)**	Continuous high local antibiotic exposure via catheters	Early clinical use in selected centers	Potential alternative to DAIR in early infections; may reduce need for revision
**VT-X7 (Phage-derived Lysin)**	Enzymatic lysis of *S. aureus* biofilm	Preclinical/early investigational	Promising adjunct for biofilm-associated infection; not yet in clinical use
**PLG0206**	Synthetic cationic peptide disrupting bacterial membranes and biofilm	Phase 1/2 clinical studies	May enhance eradication during debridement; being evaluated as adjunct to surgery

## Data Availability

No new data were created or analyzed in this study. Data sharing is not applicable to this article.

## Abbreviations

The following abbreviations are used in this manuscript:

PJI	Periprosthetic Joint Infection
TJA	Total Joint Arthroplasty
THA	Total Hip Arthroplasty
TKA	Total Knee Arthroplasty
SSI	Surgical Site Infection
CRP	C-Reactive Protein
ESR	Erythrocyte Sedimentation Rate
MSIS	Musculoskeletal Infection Society
IDSA	Infectious Diseases Society of America
ICM	International Consensus Meeting (on Musculoskeletal Infection)
CoNS	Coagulase-Negative Staphylococci
MSSA	Methicillin-Sensitive Staphylococcus aureus
MRSA	Methicillin-Resistant Staphylococcus aureus
MDRO	Multidrug-Resistant Organism
PCR	Polymerase Chain Reaction
rRNA	Ribosomal Ribonucleic Acid
mNGS	Metagenomic Next-Generation Sequencing
MALDI-TOF	Matrix-Assisted Laser Desorption Ionization–Time of Flight
EBJIS	European Bone and Joint Infection Society
IL-6	Interleukin-6
IL-7	Interleukin-7
IL-17	Interleukin-17
TNF	Tumor Necrosis Factor
LE	Leukocyte Esterase
TLR	Toll-like Receptor
MRI	Magnetic Resonance Imaging
CT	Computed Tomography
MARS-MRI	Metal Artifact Reduction Sequence Magnetic Resonance Imaging
FDG-PET/CT	Fluorodeoxyglucose Positron Emission Tomography/Computed Tomography
Hgb A1c/HbA1c	Hemoglobin A1c (Glycated Hemoglobin)
DM	Diabetes Mellitus
BMI	Body Mass Index
HEPA	High-Efficiency Particulate Air
OR	Operating Room
IV	Intravenous
DAIR	Debridement, Antibiotics, and Implant Retention
VRE	Vancomycin-Resistant Enterococcus
CPK	Creatine Phosphokinase
ID	Infectious Disease (specialist)
OVIVA	Oral Versus Intravenous Antibiotics (trial)
KLIC Score	Kidney, Liver, Index Surgery, CRP, Cement (scoring system for DAIR outcomes)
CRIME80 Score	Age >80, CRP >150, Male Sex, Infection Type (scoring system for elderly outcomes)
aPDT	Antimicrobial Photodynamic Therapy
CLAP	Continuous Local Antibiotic Perfusion
VT-X7	Vancomycin/Tobramycin Delivery Device (experimental therapy)
PLG0206	Peptilogics Antimicrobial Peptide (experimental compound)
